# Optimizing pathological diagnosis of tuberculosis: qPCR outperforms acid-fast staining in formalin-fixed paraffin-embedded tissues and enables resistance profiling

**DOI:** 10.3389/fmed.2026.1736555

**Published:** 2026-04-10

**Authors:** Yan Hu, Peng Liu, Minhui Mei, Chao Quan, Qiliang Liu

**Affiliations:** Wuhan Pulmonary Hospital, Wuhan Institute for Tuberculosis Control, Wuhan, Hubei, China

**Keywords:** acid-fast bacilli staining, formalin-fixed paraffin-embedded tissue, *Mycobacterium tuberculosis*, pathological diagnosis, qPCR

## Abstract

**Background and Objective:**

Accurate detection of *Mycobacterium tuberculosis* (MTB) in formalin-fixed paraffin-embedded (FFPE) tissues remains a significant challenge in routine pathological practice. This study aimed to evaluate the performance of the first National Medical Products Administration (NMPA)-approved quantitative polymerase chain reaction (qPCR) kit specifically optimized for FFPE tissues. We compared its efficacy with acid-fast bacilli (AFB) staining for MTB detection, while simultaneously assessing its utility for drug resistance profiling and non-tuberculous mycobacteria (NTM) identification in pathological diagnosis.

**Methods:**

We analyzed 1,050 FFPE tissue specimens that were histopathologically diagnosed as granulomatous inflammation suggestive of tuberculosis. All specimens underwent parallel testing with both qPCR (using the NMPA-approved kit) and AFB staining. Drug resistance testing was conducted on qPCR positive samples (Ct ≤ 35; *n* = 143), while Non-tuberculous Mycobacterial (NTM) identification was indicated for AFB positive/qPCR negative cases (*n* = 16).

**Results:**

The cohort, with a median age of 52 years and comprising 43.71% males, included 631 surgical specimens and 419 biopsy specimens, predominantly sourced from lung tissues (37.05%) and lymph nodes (24.67%). qPCR demonstrated a higher positive rate compared to AFB staining (63.43% vs. 26.29%, *p* < 0.001). Furthermore, qPCR exhibited a higher positive rate in surgical specimens (70.36%) compared to biopsy specimens (52.98%; *p* < 0.001) and significantly outperformed AFB staining in both lung tissues (70.44% vs. 31.62%) and lymph nodes (64.86% vs. 23.17%). The two methods displayed moderate overall concordance (58.09%; *κ* = 0.257), with the highest concordance observed in intestinal specimens (83.95%). Resistance was noted in 18.18% of cases, with 7.0% classified as multidrug-resistant tuberculosis (MDR-TB), peaking in fallopian tubes (33.33% drug-resistant TB (DR-TB), 16.67% MDR-TB). Among the 25 discordant cases, 16 successfully underwent NTM identification, revealing 4 (25.0%) cases of NTM infection.

**Conclusion:**

This study demonstrates that the qPCR kit approved by the NMPA and optimized for FFPE tissue samples exhibits high effectiveness in the pathological diagnosis of tuberculosis when culture methods are not feasible. Its performance significantly surpasses that of AFB staining. Using DNA extracted from a single FFPE tissue section, we established an integrated molecular testing strategy. This approach enables the sensitive detection of MTB through qPCR, demonstrating a higher detection rate compared to AFB staining in FFPE tissues. Furthermore, it facilitates subsequent drug resistance analysis and the differentiation of NTM species from the same DNA sample, thereby providing a practical and efficient solution for comprehensive pathological diagnosis.

## 1 Introduction

Tuberculosis is a chronic infectious disease caused by *Mycobacterium tuberculosis* (MTB) that poses a significant threat to global public health security. According to the latest global tuberculosis report released by the World Health Organization (WHO) in 2024, there were approximately 10.6 million new cases of tuberculosis worldwide in 2023, resulting in around 1.3 million deaths. Tuberculosis remains one of the leading causes of death from infectious diseases (1). The ongoing spread of drug-resistant tuberculosis, particularly rifampicin-resistant tuberculosis (RR-TB) and multidrug-resistant tuberculosis (MDR-TB), has emerged as a major challenge in global tuberculosis prevention and control efforts (2). Early and accurate microbiological diagnosis and drug susceptibility testing are critical components for achieving effective treatment and controlling the tuberculosis epidemic.

Currently, bacterial culture and culture-based phenotypic drug susceptibility testing remain the “gold standard” for diagnosing tuberculosis and drug-resistant tuberculosis (3). However, this method imposes stringent requirements regarding the number of viable bacteria in samples and their preservation conditions, involves a complex operational process, necessitates high biosafety measures, and features a detection cycle that can extend from weeks to months. These factors hinder the ability to meet the urgent clinical demand for rapid diagnosis (4). In recent years, molecular diagnostic technologies such as GeneXpert MTB/RIF and quantitative polymerase chain reaction (qPCR) have been recommended by the WHO as first-line diagnostic tools for tuberculosis and RR-TB due to their high sensitivity, specificity, and rapid detection capabilities, demonstrating robust diagnostic performance in fresh clinical samples, such as sputum (5, 6).

In clinical practice, histopathological examination remains the cornerstone for diagnosing tuberculosis, particularly in paucibacillary and extrapulmonary cases, which are often challenging to confirm bacteriologically. A typical yet non-specific histopathological manifestation of tuberculosis is granulomatous inflammation, which is characterized by epithelioid granulomas accompanied by caseous necrosis, typically surrounded by lymphocytes and Langhans giant cells (7). This specific tissue response is initiated by the host’s immune reaction to *Mycobacterium tuberculosis*. However, despite the significant indicative value of these pathological features, they are not exclusive to tuberculosis. Similar granulomatous inflammation can also be observed in various other diseases, including infections caused by non-tuberculous mycobacteria (NTM), fungi, and other pathogens, as well as non-infectious conditions such as sarcoidosis and Crohn’s disease (8). This morphological overlap presents a considerable limitation in diagnosing solely based on histopathology: while it may indicate a granulomatous disease, it does not definitively identify the underlying etiology. Therefore, an integrative approach to verifying the etiology is crucial for the accurate diagnosis of tuberculosis (5, 9).

In routine clinical settings, particularly within non-tuberculosis specialty medical institutions or during the early stages of disease when tuberculosis is not suspected, specimens are typically processed using formalin fixation and paraffin embedding (FFPE) to create slides for routine pathological examination. In contrast, fresh tissues are generally not preserved for microbiological testing. Consequently, these FFPE samples become the sole available material for subsequent retrospective or supplementary diagnoses of tuberculosis. However, the efficacy of molecular detection techniques in FFPE tissues is influenced by several factors, including formalin processing, which can lead to nucleic acid degradation, cross-linking damage, and the presence of residual inhibitors. These issues result in significant variations in detection sensitivity across different studies (10, 11).

More importantly, the optimization and systematic application of qPCR in the specific context of routine clinical FFPE tissues still involves several key aspects that warrant further exploration. First, most existing studies have a limited sample size or focus on a single type of tissue, lacking a systematic evaluation based on large-scale, FFPE samples; thus, their conclusions have limitations when extrapolated to routine pathological diagnostic practices. Second, many studies utilize generic PCR reagents, failing to adequately consider the impact of DNA degradation and cross-linking caused by FFPE processing on detection efficiency. Furthermore, there is still a relative scarcity of clinical performance data on kits that have been officially certified and specifically optimized for FFPE samples. More importantly, existing research often emphasizes comparisons of detection sensitivity and specificity, neglecting to integrate diagnosis, drug resistance analysis, and NTM differentiation within a comprehensive evaluation framework, thereby failing to fully demonstrate the overall value of this technology in modern precision pathological diagnosis.

Based on this, the present study aims to provide new evidence for optimizing the pathological molecular diagnosis of tuberculosis through the analysis of 1,050 cases of FFPE tissue samples. The focus includes three aspects: First, utilizing a large sample size that covers multiple body sites to evaluate the comprehensive performance of qPCR in diagnostic settings. Second, systematically validating the clinical efficacy of the first nucleic acid detection kit for MTB optimized for FFPE tissues, approved by the National Medical Products Administration (NMPA) of China (Beijing Xinnuomeidi Tuberculosis Mycobacterium Complex Nucleic Acid Detection Kit). Finally, exploring an integrated application model on the same technical platform that achieves the detection of pathogens, drug resistance analysis, and differentiation of non-tuberculous mycobacteria, providing practical evidence for constructing an efficient molecular diagnostic pathway for tuberculosis.

## 2 Materials and methods

### 2.1 Study subjects

**Figure 1:**
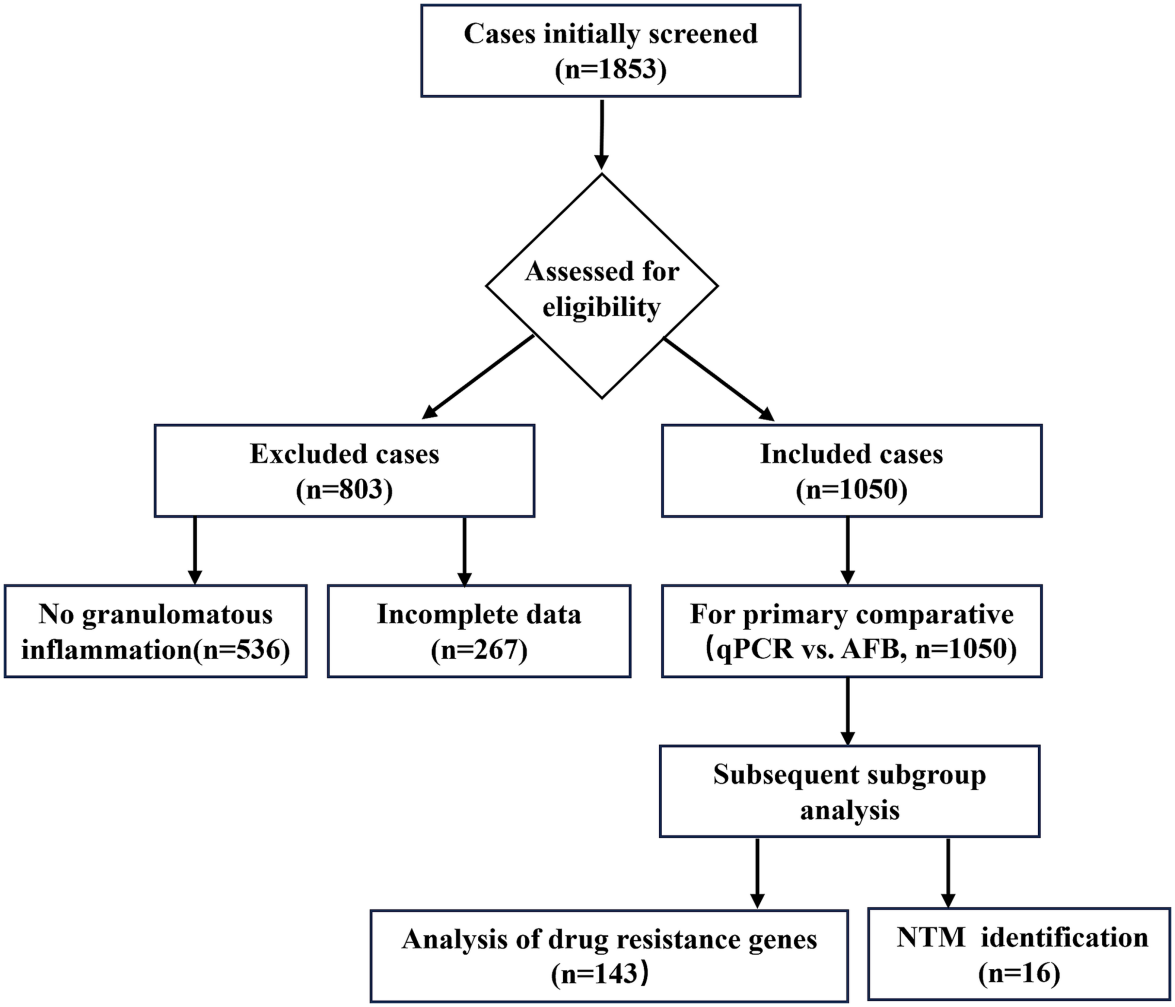
Flow diagram of case selection. Among 1,853 cases screened, 1,050 cases that met the inclusion criteria (presence of granulomatous inflammation with complete test results) were included for the primary qPCR vs. AFB analysis. Subgroup analyses included drug resistance testing (*n* = 143) and NTM identification (*n* = 16).

The detailed case selection process is illustrated in Figure 1. This study employed a prospective design to evaluate and compare diagnostic methods, using a cohort of specimens selected through retrospective review. We retrospectively analyzed 1,853 pathology consultation cases received by Wuhan Pulmonary Hospital from April 2022 to April 2025. Following an independent, double-blind review by two senior pathologists, 1,050 cases met the inclusion criteria: (1) histopathological findings indicative of chronic granulomatous inflammation with or without caseous necrosis, suggestive of tuberculosis; and (2) the availability of complete results for both standardized acid-fast staining and MTB qPCR testing. The study protocol was approved by the Medical Ethics Committee of Wuhan Pulmonary Hospital (Approval No: 2022(23)) and was conducted in accordance with the principles outlined in the Declaration of Helsinki.

### 2.2 Experimental methods

#### 2.2.1 Preparation of pathological slides

Pathological tissue sections stained with Hematoxylin and Eosin were reviewed by pathologists. In cases exhibiting pathological changes consistent with tuberculosis and possessing sufficient lesion tissue for further microbiological testing, the managing pathologist at our hospital completed a standardized application form for the preparation of blank sections. The patients then submitted this form to the pathology department of the original diagnostic institution. Subsequently, the requested routine pathological slides were prepared and returned to our hospital for analysis. Continuous sections were obtained from the most significant areas of typical lesions, such as necrosis and granulomas, with a thickness of 4–5 μm. Surgical specimens were uniformly prepared into 10 slides, while biopsy specimens were prepared into 10–15 slides. All sections underwent constant temperature baking at 56 °C for 1 h to remove paraffin and enhance tissue adhesion, thereby ensuring the stability and reproducibility of subsequent tests.

#### 2.2.2 Acid-fast staining

**Figure 2:**
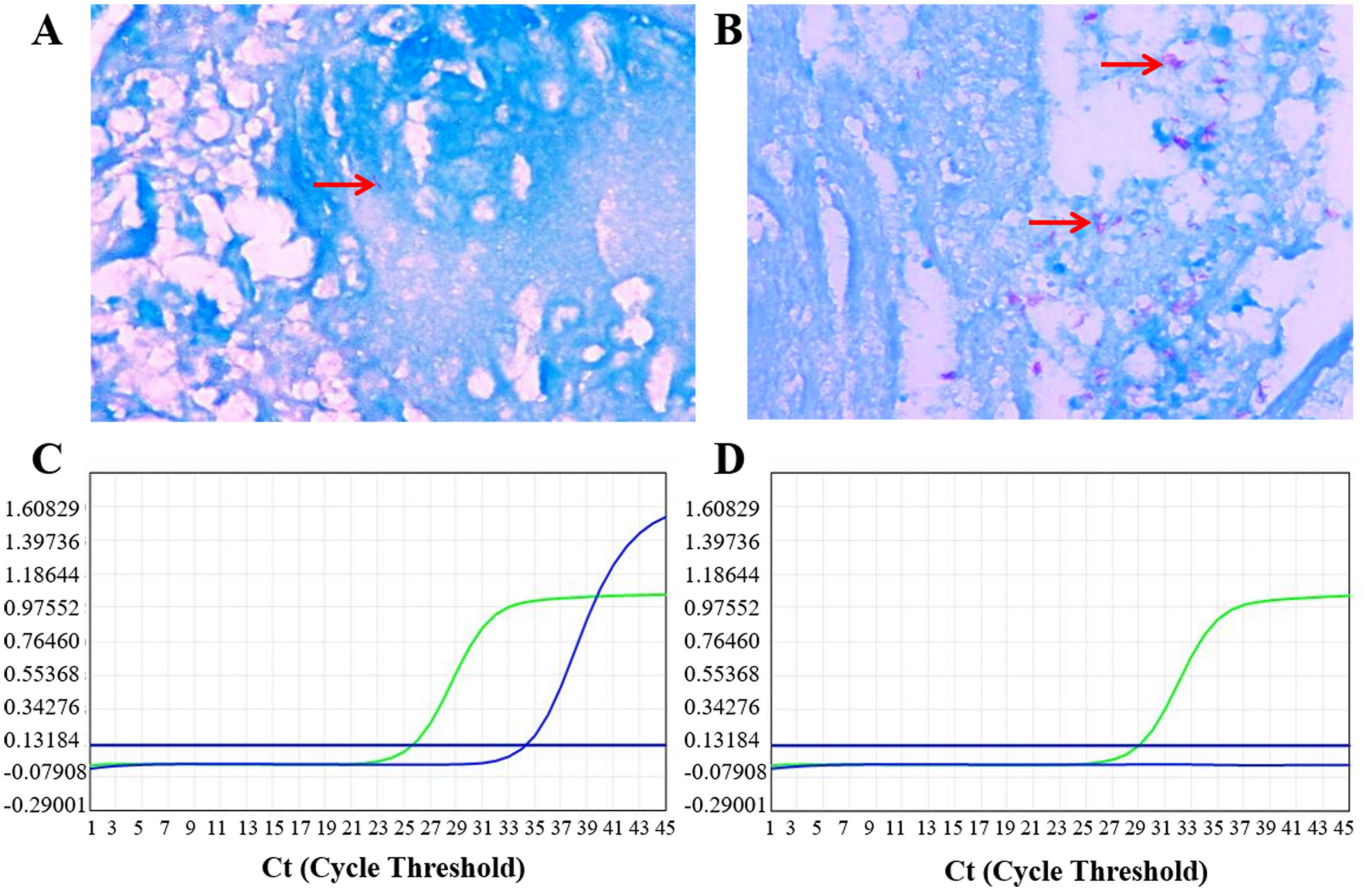
Representative results of acid-fast bacilli (AFB) staining and quantitative polymerase chain reaction (qPCR) detection of *Mycobacterium tuberculosis* (MTB) in formalin-fixed paraffin-embedded (FFPE) tissues. **(A)** Sparsely distributed acid-fast bacilli appearing as bright red, curved rod-shaped structures (indicated by red arrow). Images were acquired under an oil immersion objective at a final magnification of ×1,000. **(B)** Densely clustered acid-fast bacilli (indicated by red arrow). Images were acquired under an oil immersion objective at a final magnification of ×1,000. **(C)** Positive qPCR result: both the IS6110 target gene (blue curve) and internal control gene (green curve) show typical S-shaped amplification curves. **(D)** Negative qPCR result: no amplification of the target gene, with normal amplification of the internal control gene.

Paraffin-embedded tissue sections are dewaxed in water and subsequently subjected to acid-fast staining. The specific steps are as follows: First, apply carbol fuchsin staining solution to fully cover the tissue and allow it to stain for 30 min, ensuring that the staining solution remains undisturbed throughout this period. After staining, gently rinse the sections with deionized water for 2 min. Next, apply acid-alcohol staining solution to cover the tissue and decolorize for 1–2 min, until no red staining solution is observed. If decolorization is inadequate, wash off the acid-alcohol with running water and repeat the decolorization steps until there is no noticeable red residue in the tissue background. Following this, rinse the sections with running water for 2 min and clean with deionized water again. Then, add methylene blue counterstaining solution and counterstain for 30 s. Gently rinse from one end of the slide with running water, drain the excess water, and air dry at room temperature before mounting with neutral gum. The criteria for assessing staining results are as follows: Under an optical microscope, the tissue background should appear light blue, while acid-fast bacilli will appear bright red, typically in a curved rod shape. The number of acid-fast bacilli can be sparse (≥1 per field of view, Figure 2A) or abundant (Figure 2B).

#### 2.2.3 Microscopic imaging and analysis

Following staining, all slides were examined and photographed using an Olympus clinical bright-field microscope equipped with a Mingmei microscopic digital measurement and analysis system. Acid-fast bacilli were identified and imaged at a magnification of 1,000 × using an oil immersion objective. The stated magnification (1,000×) throughout the manuscript denotes the total optical magnification. This reporting standard, which employs magnification instead of scale bars, is conventional and appropriate for clinical pathological photomicrographs, as it accurately reflects the diagnostic viewing conditions under which pathologists conduct their assessments. All images presented are representative of the findings.

#### 2.2.4 DNA extraction and qPCR detection

Genomic DNA was extracted from FFPE tissue sections using the TIANamp FFPE DNA Kit (Tiangen Biotech, China; Cat. #DP331), in accordance with the manufacturer’s instructions. Briefly, 5 to 8 sections, each measuring 5 to 10 μm in thickness, were deparaffinized with xylene and subsequently dehydrated with absolute ethanol. Tissue lysis was conducted using Buffer GA and Proteinase K at 56 °C for one hour, followed by incubation at 90 °C for an additional hour to reverse formaldehyde cross-linking. The DNA was then purified using specific spin columns (CR2) along with buffers GD and PW, and finally eluted with 30–60 μL of pre-heated TE buffer. The extracted DNA was stored at −20 °C for subsequent analysis.

For the qPCR detection of MTB, 5 μL of the extracted DNA was used as the template in each reaction. The amplification was performed on a Shanghai Hongshi SLAN-96S fully automated medical PCR analysis system using the Beijing Xinnuomeidi Tuberculosis Mycobacterium Complex Nucleic Acid Detection Kit (NMPA Registration Permit No.: 20213401040). This TaqMan probe-based kit, which is the first NMPA-approved assay specifically designed and validated for the detection of the MTB complex in FFPE tissue specimens (including puncture biopsies and surgical tissue sections), targets the *IS6110* gene fragment along with an internal control. The PCR protocol is as follows: the reaction initiates with a 2-min incubation at 37 °C for UDG enzyme treatment to digest any potential carryover amplification products. This is followed by a 3-min pre-denaturation step at 95 °C. Subsequently, 45 amplification cycles are conducted, with each cycle comprising 15 s of denaturation at 94 °C and 35 s of annealing/extension combined with fluorescence signal acquisition at 60 °C. Finally, the instrument is cooled to 25 °C and held for 1 min.

Each detection included both positive and negative controls. The negative control (nuclease-free water) requires that both the target gene channel and the internal control channel show no amplification curves (no Ct values). The internal standard of this kit is designed to amplify only in the presence of sample DNA. Since there is no template DNA in the negative control, the lack of amplification of the internal standard is a normal phenomenon, indicating that PCR inhibition can be excluded. Conversely, the positive control necessitated the presence of S-shaped amplification curves in both channels, with Ct values below 30.0. A sample was interpreted as positive if the internal control Ct value was ≤45, and the target gene channel exhibited an S-shaped amplification curve with a Ct value ≤37; alternatively, the target gene channel could show an S-shaped amplification curve with a Ct value between 37 and 40, provided it remained ≤40 upon retesting. If the internal control Ct value exceeded 45 or was not detected, the test was deemed invalid. Typical positive and negative results are illustrated in Figures 2C,D.

#### 2.2.5 Drug resistance gene testing

For samples with qPCR Ct values ≤35, drug resistance gene testing was conducted using the Tuberculosis Drug Resistance Mutation Detection Kit from Xiamen Zhishan Biotechnology Co., Ltd. A volume of 5 μL of the extracted DNA was used as the template for this assay. This testing utilized a combination of three independent NMPA-approved kits: the Tuberculosis Mycobacterium Rifampin Resistance Mutation Detection Kit (NMPA Registration Permit No.: 20173400497), the Isoniazid Resistance Mutation Detection Kit (NMPA Registration Permit No.: 20173400485), and the Fluoroquinolone Resistance Mutation Detection Kit (NMPA Registration Permit No.: 20163401457). The test employs multiplex TaqMan probe-based high-throughput melting curve analysis technology and is performed on the LightCycler 480II system. These kits are approved for use with genomic DNA and are not limited by the source specimen type. In this study, cDNA synthesized from DNA extracted from FFPE tissues was utilized, aligning with the approved intended use. The assay primarily detects mutations in several drug resistance-related genes, including the rpoB gene associated with rifampicin resistance (detecting eight common mutation sites), the katG and inhA promoter related to isoniazid resistance, and the gyrA gene related to fluoroquinolone resistance. Each sample was analyzed in triplicate, with mutation determination criteria set at a melting temperature shift of ≥2 °C and good reproducibility.

Criteria for indeterminate results: Samples with a pre-test qPCR Ct value ≥32 were classified as “indeterminate” if they did not produce reproducible melting curves. This threshold was established through internal validation, indicating that the DNA quantities were below the assay’s reliable detection limit.

#### 2.2.6 Identification of NTM infection

For samples that are acid-fast positive but qPCR negative, the Xiamen Zhishan Mycobacterium Identification Kit (NMPA Registration Permit No.: 20223401419) was utilized to identify non-tuberculous mycobacterial species. The identification test used 5 μL of the extracted DNA as the template. This test employs the multiplex fluorescent PCR melting curve method and is conducted on the Sanity 2.0 system, facilitating the accurate differentiation of 19 clinically relevant mycobacterial species, including the *Mycobacterium tuberculosis* complex (MTBC), the *Mycobacterium avium* complex (MAC), and rapidly growing mycobacteria such as *Mycobacterium abscessus.* This kit is approved for identifying mycobacterial species from genomic DNA samples, with no restrictions on the DNA source, thereby making it suitable for use with DNA extracted from FFPE tissues in this study.

Interpretation criteria were established to ensure the validity of the test. A valid run required an internal control melting peak in the ROX channel at 78.4 °C ± 1.7 °C. The presence of mycobacteria at the genus level was indicated by a peak at 71.8 °C ± 1.6 °C in the ROX channel. A sample was confirmed as positive for MTBC if a specific melting peak was detected in the FAM channel. For NTM, confirmation required: (1) the presence of a genus-specific peak in the ROX channel, (2) a melting temperature (Tm) profile in the HEX or CY5 channels that aligns with the reference range for a specific NTM species, and (3) the absence of any signal in the MTBC-specific FAM channel. A mixed infection was defined by the presence of two or more specific melting peaks across the FAM, HEX, or CY5 channels, in addition to the genus-specific peak. A sample was classified as negative if only the internal control peak was observed. For quality control, the positive control was deemed valid only if it exhibited the expected melting peaks for MTBC in the FAM channel and for the genus in the ROX channel; otherwise, the entire experiment was considered invalid. The negative control (nuclease-free water) must show only the internal control peak; the appearance of any peak in other channels indicated potential contamination, thereby rendering the batch results unreliable.

### 2.3 Statistical analysis

Data analysis was conducted using SPSS version 22.0. Continuous data are presented as mean ± standard deviation or median (interquartile range, IQR), while categorical data are described using counts and percentages. Group comparisons were performed using the *χ*^2^ test or Fisher’s exact test when theoretical frequencies were less than five. The interpretation of Kappa values adhered to the Landis-Koch standards: 0–0.20 (slight), 0.21–0.40 (fair), 0.41–0.60 (moderate), 0.61–0.80 (substantial), and 0.81–1.00 (almost perfect). All statistical tests were two-tailed, with a *p* value of less than 0.05 considered statistically significant.

## 3 Results

### 3.1 Basic characteristics of study subjects and analysis of detection results

This study retrospectively collected 1,050 FFPE tissue specimens characterized by granulomatous inflammatory lesions from the Department of Pathology at Wuhan Pulmonary Hospital, spanning from April 2022 to April 2025. An analysis of the specimen types revealed that 631 cases (60.10%) were surgical specimens, while 419 cases (39.90%) were biopsy specimens. Among the subjects, 459 (43.71%) were male and 591 (56.29%) were female, with ages ranging from 18 to 80 years and a median age of 54 years (interquartile range: 39–65 years).

The analysis of tissue specimen sources (Table 1) revealed that lung tissue constituted the predominant source, accounting for 389 cases (37.05%), followed by lymph nodes with 259 cases (24.67%). Other sources included the intestine (81 cases, 7.71%), bone and joint tissues (58 cases, 5.52%), and the endometrium (38 cases, 3.62%). The detection results indicated that the positive rate of AFB staining was 26.29% (276 out of 1,050 specimens), whereas the positive rate of qPCR was significantly higher at 63.43% (666 out of 1,050 specimens). Among the 25 specimens that tested positive for AFB but negative for qPCR, mycobacterial species identification was successfully performed for 16 specimens, which identified 4 cases (25.00%) of NTM infection: 2 cases of *Mycobacterium gordonae* and 1 case each of *Mycobacterium intracellulare* and *Mycobacterium fortuitum*. The remaining 9 cases were not tested, as the required patient informed consent (including agreement to the associated cost) for this assay could not be obtained in this study.

**Table 1:** General characteristics of the population.

Variables	Value, *n* (%) or median (IQR)
Age (yr)	54 (39–65)
Gender, *n* (%)	
Male	459 (43.71)
Female	591 (56.29)
Specimen type, *n* (%)	
Surgical	631 (60.10)
Biopsy	419 (39.90)
Primary tissue source, *n* (%)	
Lung	389 (37.05)
Lymph node	259 (24.67)
Intestine	81 (7.71)
Bone/joint	58 (5.52)
Endometrium	38 (3.62)
Detection methods, *n* (%)	
AFB positive	276 (26.29)
qPCR positive	666 (63.43)

Surgical, Surgical specimen; Biopsy, Biopsy specimen; IQR, interquartile range; AFB, acid-fast bacilli; qPCR, quantitative polymerase chain reaction.

### 3.2 Comparison of AFB staining and qPCR results based on differences in lesion origin and tissue type

**Figure 3:**
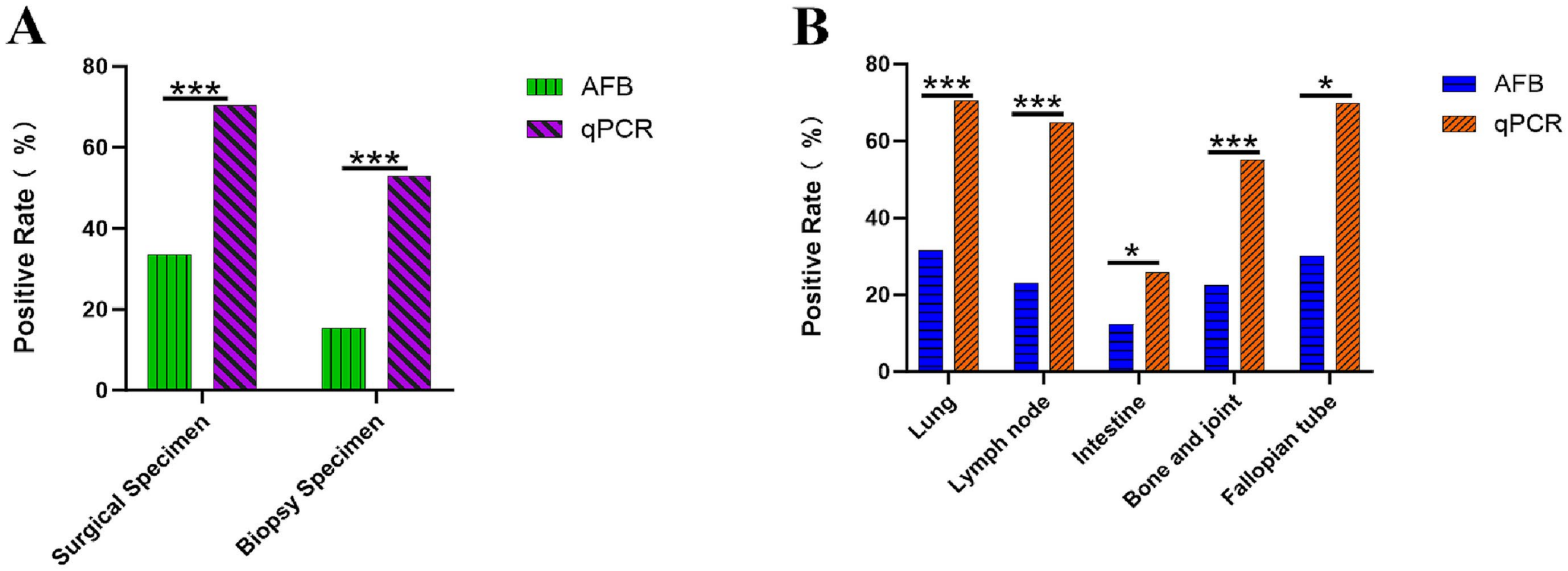
Positive rates of *Mycobacterium tuberculosis* by acid-fast bacilli staining and quantitative polymerase chain reaction **(A)** Positive rates in surgical and biopsy specimens. **(B)** Positive rates across different tissue types (Lung, Lymph node, Intestine, Bone and joint, Fallopian Tube).

Among the 1,050 specimens, the overall positive rate of AFB staining was 26.29% (276/1,050), which was significantly lower than the 63.43% (666/1,050) detected by qPCR (*χ*^2^ = 292.812, *p* < 0.001). Analyzing by specimen type (Figure 3A), the AFB positivity rate was higher in surgical specimens (33.60%, 212/631) compared to biopsy specimens (15.27%, 64/419). In contrast, the qPCR positivity rates were 70.36% (444/631) in surgical specimens and 52.98% (222/419) in biopsy specimens, with both differences being statistically significant (all *p* < 0.001). Further analysis of tissue types with larger sample sizes revealed that in lung tissue (*n* = 389), the AFB positivity rate was 31.62% (123/389), while the qPCR positivity rate reached 70.44% (274/389). In lymph node tissue (*n* = 259), the AFB positivity rate was 23.17% (60/259), and the qPCR positivity rate was 64.86% (168/259). Both differences were statistically significant (*p* < 0.001). Additionally, results from other tissue types (such as intestinal, bone and joint, and endometrium) demonstrated that qPCR generally exhibited higher positivity rates than AFB (Table 2, Figure 3B). In summary, the detection sensitivity of qPCR was significantly superior to that of AFB staining across all tissue types.

**Table 2:** Comparison of AFB staining and qPCR detection rates across different tissue sources (*N* = 1,050).

Tissue source	Specimen type	Total (*n*)	AFB positive [%(*n*)]	qPCR positive [%(*n*)]	*χ*^2^ value	*p* value
Lung	Surgical	219	41.55 (91)	77.17 (169)	57.580	<0.001
	Biopsy	170	18.82 (32)	61.76 (105)	65.149	<0.001
	Subtotal	389	31.62 (123)	70.44 (274)	117.278	<0.001
Lymph node	Surgical	165	33.33 (55)	70.30 (116)	45.163	<0.001
	Biopsy	94	5.32 (5)	55.32 (52)	55.617	<0.001
	Subtotal	259	23.17 (60)	64.86 (168)	91.379	<0.001
Intestine	Surgical	6	50.00 (3)	66.67 (4)	/	1.000
	Biopsy	75	9.33 (7)	22.67 (17)	4.960	0.026
	Subtotal	81	12.35 (10)	25.93 (21)	4.827	0.028
Bone and joint	Surgical	49	24.49 (12)	57.14 (28)	10.814	0.001
	Biopsy	9	11.11 (1)	44.44 (4)	/	0.294
	Subtotal	58	22.41 (13)	55.17 (32)	13.107	<0.001
Endometrium	Surgical	38	26.32 (10)	68.42 (26)	13.511	<0.001
	Biopsy	0	0.00	0.00	/	/
	Subtotal	38	26.32 (10)	68.42 (26)	13.511	<0.001
Pleura	Surgical	4	25.00 (1)	75.00 (3)	/	0.486
	Biopsy	22	40.91 (9)	68.18 (15)	3.300	0.069
	Subtotal	26	38.46 (10)	69.23 (18)	4.952	0.026
Peritoneum/abdominal wall	Surgical	16	31.25 (5)	75.00 (12)	6.149	0.013
	Biopsy	5	0.00	80.00 (4)	/	0.048
	Subtotal	21	23.81 (5)	76.19 (16)	11.524	<0.001
Soft tissue	Surgical	15	13.33 (2)	60.00 (9)	70.330	0.008
	Biopsy	3	0.00	0.00	/	/
	Subtotal	18	11.11 (2)	50.00 (9)	6.415	0.011
Skin	Surgical	19	15.79 (3)	15.79 (3)	0.000	1.000
	Biopsy	0	0.00	0.00		
	Subtotal	19	15.79 (3)	15.79 (3)	0.000	1.000
Fallopian tube	Surgical	20	6.00	70.00 (14)	6.400	0.011
	Biopsy	0	0.00	0.00	/	/
	Subtotal	20	30.00 (6)	70.00 (14)	6.400	0.011
Chest wall	Surgical	10	3.00	80.00 (8)	5.051	0.025
	Biopsy	7	0.00	28.57 (2)		0.462
	Subtotal	17	17.65 (3)	58.82 (10)	6.103	0.013
Larynx/pharynx	Surgical	7	28.57 (2)	71.43 (5)	/	0.286
	Biopsy	10	50.00 (5)	80.00 (8)	/	0.350
	Subtotal	17	41.18 (7)	76.47 (13)	4.371	0.037
Testis/epididymis	Surgical	11	36.36 (4)	72.73 (8)	2.933	0.087
	Biopsy	0	0.00	0.00	/	/
	Subtotal	11	36.36 (4)	72.73 (8)	2.933	0.087
Other	Surgical	52	28.85 (15)	75.00 (39)	22.187	<0.001
	Biopsy	24	20.83 (5)	62.50 (15)	8.571	0.003
	Subtotal	76	26.32 (20)	71.05 (54)	30.442	<0.001
Overall	Surgical	631	33.60 (212)	70.36 (444)	170.867	<0.001
	Biopsy	419	15.27 (64)	52.98 (222)	132.511	<0.001
	Total	1,050	26.29 (276)	63.43 (666)	292.812	<0.001

Statistical comparisons were made by *χ*^2^ test (with Yates’ correction) or Fisher’s exact test (for expected frequencies <5). Other tissues included: liver, adrenal, kidney, ear, mediastinum, esophagus, salivary gland, brain, pancreas, abdominal mass, prostate, oral cavity, bladder, breast, and stomach. AFB, acid-fast bacilli; qPCR, quantitative polymerase chain reaction; Surgical, surgical specimen; Biopsy, biopsy specimen.

### 3.3 Agreement analysis between AFB staining and qPCR for detecting *Mycobacterium tuberculosis*

An agreement analysis was performed to compare the results of AFB staining and qPCR across 1,050 clinical specimens. The overall agreement between the two methods was 58.09% (610/1,050), calculated as the proportion of specimens with concordant results (both tests positive or both negative) among the total specimens (*κ* = 0.257, *p* = 0.021; Table 3). The Kappa value suggested a slight agreement between the two methods, and the difference was statistically significant.

**Table 3:** Consistency analysis of AFB staining and qPCR for MTB detection.

Category	qPCR positive, *n* (%)	qPCR negative, *n* (%)	Total, *n* (%)
AFB positive	251 (23.90)	25 (2.38)	276 (26.29)
AFB negative	415 (39.52)	359 (34.19)	774 (73.71)
Total	666 (63.43)	384 (36.57)	1,050 (100.00)
Kappa value	0.257		
*p* value	0.021		

AFB, acid-fast bacilli; qPCR, quantitative polymerase chain reaction; MTB, *Mycobacterium tuberculosis*.

**Figure 4:**
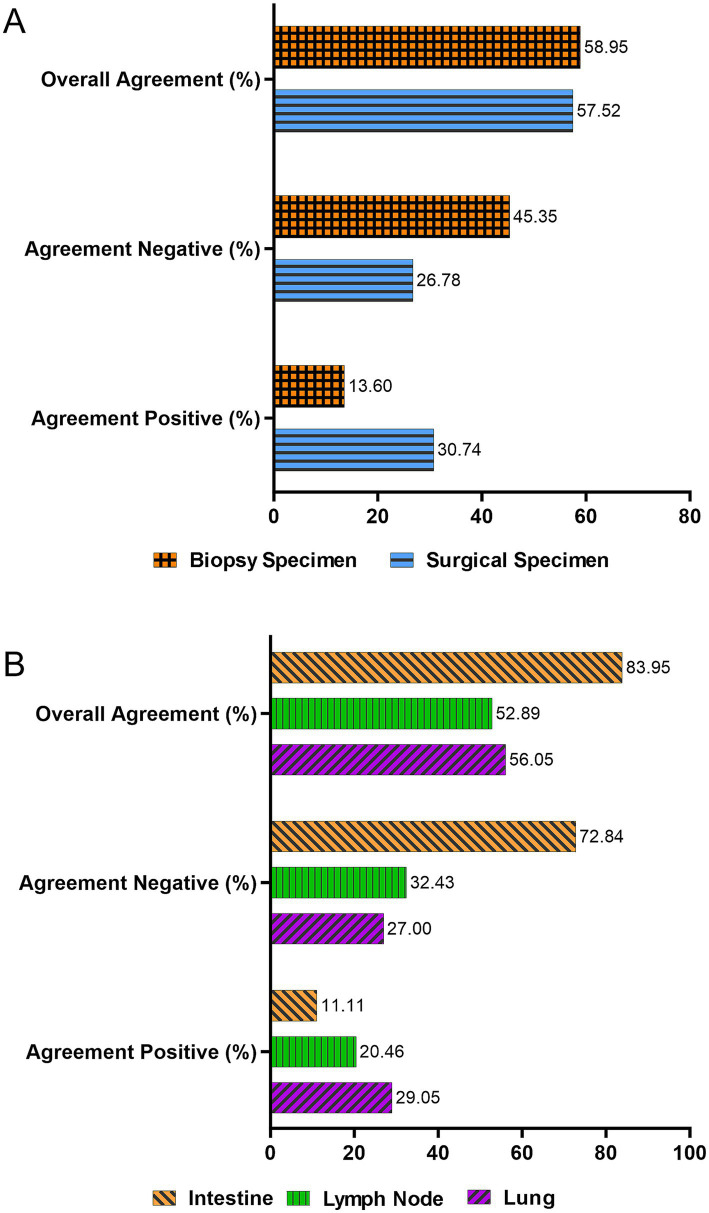
Agreement analysis between acid-fast bacilli (AFB) staining and quantitative polymerase chain reaction (qPCR) for detecting *Mycobacterium tuberculosis* (MTB) across different specimen types and tissue sources. **(A)** Agreement rates between AFB and qPCR for MTB detection, categorized by specimen type. **(B)** Agreement rates across different tissue sources (e.g., lung, lymph node, intestine). The overall agreement for each category was calculated as the proportion of specimens where both test results were concordant (both positive or both negative).

Analysis by specimen type (Figure 4A) revealed an agreement rate of 57.52% (363/631) in surgical specimens (*κ* = 0.251, *p* < 0.001) and 58.95% (247/419) in biopsy specimens (*κ* = 0.211, *p* = 0.002) The Kappa values for both surgical and biopsy specimens indicated fair agreement, and the results were statistically significant. When analyzed by tissue source (Figure 4B), the agreement rate between AFB and qPCR was 56.05% (218/389) in lung tissue, 52.89% (137/259) in lymph node tissue, and 83.95% (68/81) in intestinal tissue. The agreement in intestinal tissue was significantly higher than in lung and lymph node tissues (*p* < 0.001), indicating the best agreement among the evaluated tissue types.

### 3.4 Drug resistance analysis of *Mycobacterium tuberculosis*

Among the 666 specimens that tested positive by qPCR, 143 (21.47%) with Ct values ≤35 were subjected to drug resistance gene detection. The results indicated that sensitive strains accounted for 71.33% (102/143), while drug-resistant strains constituted 18.18% (Table 4). Distinct differences in resistance profiles were observed across specimen sources. Bone and joint specimens exhibited the highest sensitivity rate (88.89%, 8/9), followed by lung tissue (74.63%, 50/67) and lymph nodes (70.00%, 21/30). In contrast, fallopian tube specimens demonstrated the lowest sensitivity rate (16.67%, 1/6) and a comparatively high drug resistance rate, with drug-resistant tuberculosis (DR-TB) at 33.33% and multidrug-resistant tuberculosis (MDR-TB) at 16.67%. DR-TB was detected in 11.89% (17/143) of the specimens overall, with the majority found in lung tissue specimens (13.43%, 9/67). MDR-TB accounted for 6.99% (10/143) of cases and was detected at higher rates in lung tissue and fallopian tube specimens (5.97 and 16.67%, respectively). Additionally, some specimens yielded indeterminate results due to insufficient DNA concentration. The drug resistance profile of other specimen types (*n* = 31) was generally consistent with the overall trend, showing a sensitivity rate of 70.97%, with both DR-TB and MDR-TB detected at 9.68%.

**Table 4:** Drug resistance profiles across specimen types.

Category	Total (*N* = 143)	Lung (*N* = 67)	Lymph node (*N* = 30)	Bone and joint (*N* = 9)	Fallopian tube (*N* = 6)	Other specimens (*N* = 31)
DS-TB	102 (71.33%)	50 (74.63%)	21 (70.00%)	8 (88.89%)	1 (16.67%)	22 (70.97%)
DR-TB	17 (11.89%)	9 (13.43%)	3 (10.00%)	0 (0.00%)	2 (33.33%)	3 (9.68%)
MDR-TB	10 (6.99%)	4 (5.97%)	0 (0.00%)	1 (11.11%)	1 (16.67%)	3 (9.68%)
Indeterminate	15 (10.49%)	4 (5.97%)	6 (20.00%)	0 (0.00%)	2 (33.33%)	3 (9.68%)

DS-TB, drug-susceptible tuberculosis; DR-TB, drug-resistant tuberculosis; MDR-TB, multidrug-resistant tuberculosis. DS-TB was defined as infection with a *Mycobacterium tuberculosis* strain susceptible to all first-line anti-tuberculosis drugs; DR-TB as resistance to any one of rifampicin, isoniazid, or a fluoroquinolone; and MDR-TB as concurrent resistance to at least rifampicin and isoniazid. Other specimens included nasopharynx, liver, brain, salivary gland, testis/epididymis, adrenal gland, intestine, soft tissue, pancreas, mediastinum, larynx/pharynx, chest wall, pleura, endometrium. Indeterminate results were due to insufficient DNA concentration for reliable interpretation. Among the 10 MDR-TB cases, one case (from lung tissue) demonstrated additional resistance to a fluoroquinolone.

## 4 Discussion

The definitive diagnosis of tuberculosis necessitates the integration of pathological changes with etiological evidence. Characteristic pathological changes, such as epithelioid granulomas with caseous necrosis, while suggestive of tuberculosis, are not exclusive to this disease and may also manifest in various conditions, including fungal infections, sarcoidosis, and NTM disease (12). Consequently, the “Chinese Expert Consensus on the Pathological Diagnosis of Tuberculosis” underscores that the presence of MTB must be confirmed through etiological testing based on morphological findings to establish a definitive diagnosis (13). Among the various methods for etiological detection, PCR-based molecular detection technology has emerged as a crucial adjunct for etiological identification due to its high sensitivity and specificity. Particularly in situations where bacterial culture is not feasible, molecular diagnostic techniques demonstrate unique value (4, 5). Although acid-fast staining remains widely utilized because of its straightforward procedure, low cost, and capacity to visually identify MTB within histomorphological contexts, its sensitivity is limited, and its recognition rate is particularly low among pathologists who do not specialize in tuberculosis. Furthermore, acid-fast staining cannot differentiate between MTB and NTM, which further restricts its specificity (14). Therefore, PCR, as a rapid and highly sensitive molecular detection method, has demonstrated significant value in detecting MTB. By specifically amplifying target gene sequences of MTB, it can effectively distinguish MTB from NTM, providing crucial molecular evidence for the differential diagnosis of granulomatous diseases (15–17).

This study, through large-sample analysis, confirms the superior performance of a qRT-PCR kit optimized for FFPE tissues compared to acid-fast staining. An analysis of 1,050 FFPE samples from patients with suspected tuberculosis characterized by granulomatous lesions revealed an overall MTB positivity rate of 63.43% by qPCR, significantly higher than the 26.29% positivity rate by acid-fast staining. This result not only aligns with the general principle that molecular detection techniques offer higher sensitivity than traditional microscopic methods (4, 18) but, more importantly, this positivity rate exceeds those reported in most previous studies using universal MTB qPCR kits on sputum or fresh tissue (19). This highlights the unique value of this specially optimized kit for FFPE samples and its great potential in retrospective diagnoses when fresh specimens are unavailable. This study enhances the existing understanding across three dimensions. First, leveraging multi-site, large-sample clinical data, it elucidates the actual efficacy spectrum of qPCR across various types of clinical specimens, including surgical and biopsy samples, as well as specialized samples from bones and joints. This provides empirical evidence for the development of differentiated diagnostic strategies. Second, by validating the first domestically optimized reagent kit specifically designed for FFPE, the study confirms the critical role of targeted technical improvements in increasing the positive detection rate, particularly in low bacterial load samples such as biopsies. Third, an integrated evaluation pathway of “Pathogen Detection–Drug Resistance Analysis-NTM Identification” has been established, demonstrating how molecular techniques can systematically address a series of clinical diagnostic questions: “Are there bacteria? Are they drug-resistant? What type of bacteria are present?” This offers a novel perspective for optimizing the pathological diagnosis process of tuberculosis.

In-depth analysis revealed that the detection results were significantly influenced by specimen type and tissue origin. This study confirmed that the positivity rates of both qPCR and acid-fast staining were higher in surgical resection specimens compared to biopsy specimens. This discrepancy may stem from the limited tissue quantity in biopsies, typically lower bacterial loads, and tissue consumption from prior HE and immunohistochemical staining, all of which increase the difficulty of successful DNA extraction and PCR amplification. Therefore, it is recommended to prioritize the most representative areas of lesions within biopsy tissue. In our pathological diagnostic practice, we have observed that necrotic areas exhibit the highest bacterial load and are most suitable for preparing blank sections for subsequent etiological testing. It is advisable to prepare 10–15 blank sections to ensure optimal DNA quality. Nonetheless, the optimized kit still achieved a 52.98% detection rate in the highly challenging biopsy samples, highlighting its practical value in clinical scenarios with limited tissue availability, offering the possibility of precise diagnosis for many patients from whom surgical specimens cannot be obtained.

Further analysis revealed that detection efficiency varied across different tissue types. In samples with large sizes, such as lung and lymph node tissues, the qPCR positivity rates were 70.44 and 64.86%, respectively, while the acid-fast staining positivity rates were 31.62 and 23.17%, respectively. This indicates a higher MTB load in lung tissues, consistent with previous studies suggesting that extrapulmonary tuberculosis typically has a lower bacterial load (20, 21). In contrast, bone and joint tissues, decalcification is required, and severe DNA damage results in a relatively low qPCR positivity rate of 55.17%. This can be attributed to the strong decalcifying agents commonly used in practice, such as nitric acid or hydrochloric acid. These agents not only hydrolyze the lipid-rich cell wall of MTB, compromising its structural integrity and leading to false-negative acid-fast bacilli staining, but they also cause significant degradation of bacterial DNA, thereby reducing qPCR sensitivity (11). Furthermore, acid residues may inhibit enzyme activity in the qPCR reaction. To address this issue, we recommend optimizing the decalcification protocol for molecular detection. It is preferable to use mild chelating agents, such as ethylenediaminetetraacetic acid (EDTA), as they preserve nucleic acid integrity through gentle chelation of calcium ions rather than corrosive hydrolysis. Practical measures to enhance the decalcification efficacy of EDTA include slicing specimens to increase surface area, conducting the decalcification process at 37 °C, frequently changing the decalcifying solution, and thoroughly rinsing post-decalcification (for example, rinsing under running water for more than 1 h) to reduce residual inhibitors. This strategy requires further practical validation. Studies have confirmed that compared to strong acids, EDTA decalcification better preserves nucleic acids, significantly improving the success rate of downstream molecular detection (22, 23). This strategy requires further practical validation.

In this study, the concordance rate between qRT-PCR and acid-fast staining was 58.09% (Kappa = 0.257). This discrepancy precisely reveals the deeper advantages of molecular detection technology. This discrepancy arises primarily from two factors: firstly, qPCR technology possesses higher sensitivity, enabling it to detect samples with low bacterial loads that are undetectable by acid-fast staining; secondly, acid-fast staining cannot differentiate between MTB and NTM, which may result in false discrepancies. In the NTM identification performed on AFB-positive but qRT-PCR (MTBC-specific) negative cases in this study, 25% (4/16) were confirmed as NTM infections. This finding is crucial because NTM infections can cause granulomatous lesions extremely similar to tuberculosis, but treatment strategies and prognoses differ significantly. Misdiagnosis may lead to treatment failure or drug resistance (24, 25). The results of this study resonate with the clinical utility of PCR for differential diagnosis of tuberculosis and NTM infection in paraffin-embedded lung tissues reported by Kim et al. (10). Therefore, when significant discrepancies occur between the two methods, conducting NTM differential diagnosis is highly clinically necessary. The integrated pathway of “Pathogen Detection–Discrepancy Analysis-NTM Identification” established in this study elevates the value of molecular detection from mere pathogen detection to precise pathogen identification.

While fully affirming the advantages of qPCR, it is also essential to objectively recognize its limitations and the complementary value of traditional acid-fast staining (26). A fundamental limitation of qPCR is its inability to distinguish DNA originating from viable versus dead bacteria. A positive result only indicates the presence of MTB DNA and does not necessarily imply active infection, as it could also stem from past healed infections or residual nucleic acids (18). Therefore, the interpretation of qRT-PCR results must be combined with histopathological evidence of active lesions (such as necrotizing granulomas). In contrast, the unique advantage of acid-fast staining lies in its ability to directly and visually demonstrate intact bacterial morphology within the histomorphological context, allowing assessment of bacterial load in the tissue and its spatial relationship with the host inflammatory response-information lost in DNA extraction-based qPCR (27). Therefore, the ideal diagnostic strategy should be a combination of morphology and molecular technology, not substitution. Integrating acid-fast staining, which provides direct morphological evidence, with qPCR technology, which provides highly sensitive and specific etiological evidence, can establish a more comprehensive and reliable foundation for the precise pathological diagnosis of tuberculosis.

Furthermore, the integrated detection pathway constructed in this study successfully enabled the detection of drug resistance-related genes. The results of drug resistance gene detection indicated that among 143 qPCR positive samples, the overall drug resistance rate was 18.18%, with MDR-TB accounting for 6.99%. Notably, the drug resistance rate of fallopian tube tuberculosis was significantly higher than that of other types. This suggests potential differences in the microenvironment or drug permeability of lesions at different sites, leading to “spatial heterogeneity” of drug resistance. This finding provides clues for developing more precise anti-tuberculosis treatment regimens for tuberculosis at specific sites (28). In contrast, bone and joint samples exhibited a relatively high sensitivity of 88.89%, which may be attributed to the lower bacterial load and reduced selective pressure for mutations in such lesions. As all samples were FFPE tissues, traditional bacterial culture and phenotypic drug susceptibility testing were not possible. Thus, molecular drug susceptibility testing based on FFPE samples became a reliable way to obtain drug resistance information (29). This further demonstrates the great potential of this integrated detection strategy in guiding clinical treatment, especially for extrapulmonary tuberculosis and chronic cases where culture is not feasible. Our results confirm that even in challenging samples like FFPE, molecular drug susceptibility testing can provide critical drug resistance information, consistent with the WHO’s strategic direction recommending molecular techniques for the rapid diagnosis of drug-resistant tuberculosis (5).

This study acknowledges several limitations that must be considered. Firstly, and most importantly, the primary limitation stems from the nature of our study material: the absence of bacterial culture as the diagnostic “gold standard.” As all specimens were FFPE tissues, culture is technically infeasible due to formalin fixation, which kills bacteria and cross-links nucleic acids. The inability to culture FFPE tissue samples, combined with the etiological heterogeneity of the cases of granulomatous inflammation included in the study, precludes the calculation of classical diagnostic indicators such as sensitivity and specificity for qPCR and AFB staining. Consequently, this study utilizes “positive rate” and “agreement analysis” as alternative metrics. The results should be interpreted as a comparative evaluation of the performance of the two detection methods, rather than as an absolute assessment of diagnostic accuracy for a specific pathogen. Secondly, certain specimens, particularly those requiring decalcification (e.g., bone tissue), were undetectable, likely due to DNA degradation. Thirdly, utilizing a Ct value of ≤35 as the threshold for drug resistance testing, while ensuring the reliability of results according to the specifications of the kit, may inadvertently exclude certain low-bacterial-load samples from resistance profiling. Additionally, this study did not include a direct comparison with established molecular methods, such as line probe assays, primarily due to limitations arising from the limited quantity and fragmented nature of DNA extracted from archived FFPE samples. Future prospective validations should focus on addressing these comparisons, as they represent a significant avenue for further research. Fourthly, the kit used in this study is the first specifically optimized for FFPE tissue samples; there is currently no internationally recognized, directly comparable FFPE tissue detection kit. Its performance needs further validation in broader prospective studies. Finally, regarding drug resistance, while phenotypic Drug Susceptibility Testing was not possible, the molecular resistance profiling conducted on DNA from the same FFPE samples represents the only feasible approach to obtain resistance information in this context, aligning with WHO recommendations for the rapid molecular diagnosis of drug-resistant TB.

## 5 Conclusion

Based on an analysis of 1,050 FFPE samples where culture was not feasible, this study provides the first systematic validation of China’s inaugural qPCR kit specifically optimized for FFPE tissues. The results demonstrate that this technology exhibits a significantly higher detection rate compared to acid-fast staining. Furthermore, our findings indicate that DNA extracted from FFPE tissues can be effectively utilized not only for sensitive MTB detection but also for subsequent drug resistance analysis and NTM differentiation when adequate DNA quality is achieved. The study revealed notable variations in detection performance across different sample types, with surgical specimens showing higher positivity rates than biopsy specimens. Additionally, the observed drug resistance patterns, particularly the elevated resistance rate in fallopian tube tuberculosis, highlight the importance of resistance monitoring in extrapulmonary tuberculosis. This comprehensive evaluation supports the clinical utility of this qPCR technology as a valuable adjunct in the pathological diagnosis of tuberculosis, offering a feasible approach for integrating molecular detection with resistance profiling and species identification within routine diagnostic workflows.

## Data Availability

The original contributions presented in the study are included in the article/Supplementary material, further inquiries can be directed to the corresponding author.
