# Supplementary figures and images for: Optimizing pathological diagnosis of tuberculosis: qPCR outperforms acid-fast staining in formalin-fixed paraffin-embedded tissues and enables resistance profiling
Source: Front Med (Lausanne). 2026 Apr 10;13:1736555. doi: 10.3389/fmed.2026.1736555 (PMC13106081; doi:10.3389/fmed.2026.1736555)

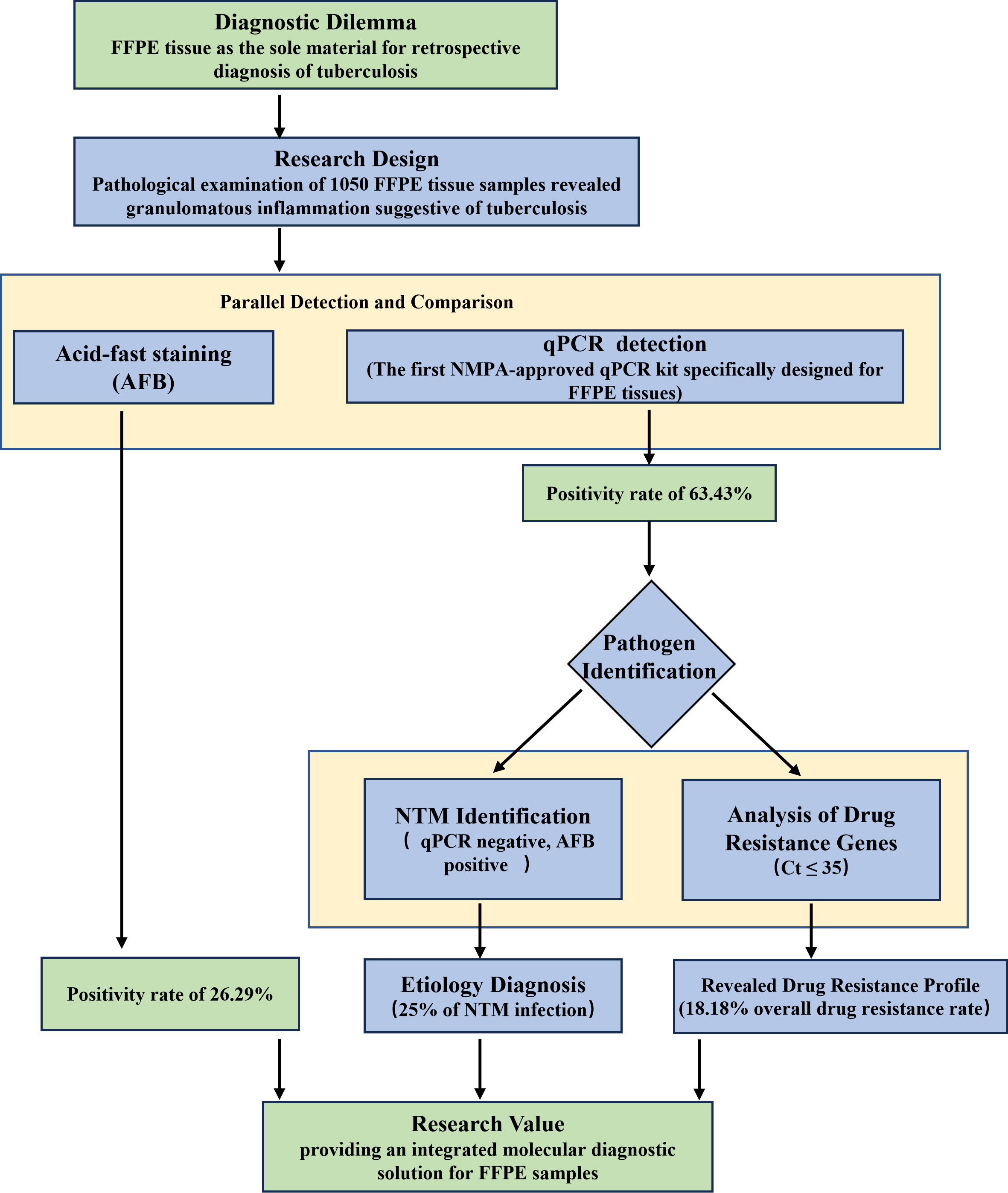

Supplement: Supplementary file 1 [file Image_1.tif]
